# Regulation of cytokine gene expression by orosomucoid in neonatal swine adipose tissue

**DOI:** 10.1186/s40104-016-0081-0

**Published:** 2016-04-14

**Authors:** Timothy G. Ramsay, Margo J. Stoll, Le Ann Blomberg, Thomas J. Caperna

**Affiliations:** Animal Biosciences and Biotechnology Laboratory, USDA/ARS, Beltsville Agricultural Research Center, USDA, Beltsville, MD 20705 USA

**Keywords:** Adipose, Cytokines, Neonate, Orosomucoid, Swine

## Abstract

**Background:**

Porcine adipose tissue expresses orosomucoid (ORM1) mRNA, a protein with anti-inflammatory and immunomodulatory properties. Previous research has demonstrated that porcine ORM1 can reduce insulin stimulated glucose metabolism in porcine adipose tissue *in vitro*. The present study was designed to examine the preweaning ontogeny of ORM1 mRNA abundance in porcine subcutaneous adipose and to determine if ORM1 can regulate mRNA abundance of inflammatory cytokines that contribute to insulin resistance in primary cultures derived from neonatal porcine subcutaneous adipose tissue. Cultures were differentiated *in vitro* and subsequently the adipocyte containing cultures were incubated for 24 h with 0–5000 ng porcine ORM1/mL medium. Cultures were then harvested, total RNA extracted for use in reverse transcription and the mRNA abundance of cytokine mRNA quantified by real-time PCR.

**Results:**

ORM1 mRNA abundance within neonatal adipose tissue does not change from d 1 to d 21 of age and is a very small fraction relative to liver mRNA abundance. The ORM1 mRNA level in porcine adipocytes and stromal-vascular cells are similar (*P* > 0.05). Treatment with ORM1 did not affect TNFα (tumor necrosis factor α) mRNA level (*P* > 0.05), while interleukin 6 (IL6) mRNA abundance was reduced 32 % at 1,000 ng ORM1/mL (*P* < 0.01). However, TNFα protein content in the cell culture media was reduced by ORM1 treatment (5,000 ng/mL, *P* < 0.05), whereas ORM1 had no detectable effect on the media content of IL6 (*P* > 0.05). The reduction of macrophage migration inhibitory factor (MIF) mRNA abundance by ORM1 was dose dependent (*P* < 0.01). Monocyte chemotactic protein (MCP) mRNA level was reduced 27 % by 1,000 ng ORM1/mL (*P* < 0.05).

**Conclusions:**

The data suggest that ORM1 has limited effects TNFα, IL6, MIF or MCP expression at the concentrations tested. Secondly, these cytokines do not appear to contribute to the reported insulin resistance induced by ORM1 in porcine adipose tissue *in vitro* as an increase in the abundance of these inflammatory cytokines would be predicted during an insulin resistant state.

## Background

Orosomucoid (ORM1) is an acute phase protein (APP) and has some anti-inflammatory and immunomodulatory properties [1, 2]; it is also known as α1-acid glycoprotein. However, ORM1 in swine appears to be a negative acute phase protein, unlike in other species that have been evaluated [3, 4]. Orosomucoid is the most abundant protein in the serum of the newborn pig [5, 6]. The liver is considered the major site of ORM1 expression in most species, but the mRNA has been detected in several other tissues [7]. Recently, Rødgaard et al. [8] reported ORM1 gene expression by subcutaneous and visceral adipose tissues. However, there is no information on the ontogeny of ORM1 gene expression in the adipose tissue during early postnatal development during the time of rapid changes in serum ORM1 protein [5] and liver ORM1 mRNA level [9]. This became one objective of the present study.

Adipose tissue expression of ORM1 or the actions of hepatic secreted ORM1 within adipose tissue may be of importance due to the association of APPs with metabolic syndrome and insulin resistance in animal models of obesity [10]. The presence of APPs has been associated with the expression of numerous cytokines within the adipose tissue [11, 12], including tumor necrosis factor α (TNFα), interleukin 6 (IL6) and monocyte chemotactic protein (MCP). These cytokines have all been demonstrated to contribute to insulin resistance [10–12]. These inflammatory cytokines are expressed in neonatal porcine adipose tissue but their regulation has not been examined [13]. The potential role of ORM1, among these APPs, in regulating the expression of TNFα, IL6 or other inflammatory cytokines regulated by TNFα within the adipose tissue has not been investigated. This is of current relevance as orosomucoid 1 has recently been shown to reduce insulin stimulated glucose utilization by porcine adipose tissue [14]. The present study was designed to determine if ORM1 can stimulate inflammatory cytokine gene expression in porcine adipose tissue which could contribute to the reported insulin resistance produced by porcine ORM1.

## Methods

### Ontogeny

The first experiment was performed to characterize the ontogeny of adipose tissue ORM1 mRNA abundance during the preweaning period. Selected crossbred female pigs (Yorkshire × Landrace × Poland China; *n* = 5) were vigorous and healthy. All procedures performed in these studies involving animals were in accordance with the ethical standards of the institutional Animal Care and Use Committee and were approved by the committee. They were visually inspected for cuts, scrapes or any sign of poor health prior to use in this experiment. Pigs were not littermates. Dorsal subcutaneous adipose tissue was collected from between the second and fourth thoracic vertebrae of 1, 7 or 21 d old pigs following i.v. pentobarbital sodium administration (200 mg/kg BW). Euthanasia was performed in accordance with the ethical standards of the institutional Animal Care and Use Committee and were approved by the committee. Liver samples were collected from the same pigs. Tissue was rinsed in ice-chilled PBS, blotted, diced, frozen in liquid N_2_ and then stored at −80 °C for extraction of total RNA at a later date. These total RNA samples were then used in absolute quantitative PCR to permit comparison of ORM1 expression between adipose and liver tissues.

### Adipocyte and stromal-vascular cell isolation

A second experiment was performed to determine the source of ORM1 within adipose tissue. Dorsal subcutaneous adipose tissue was collected from between the second and fourth thoracic vertebrae of 21 d old pigs following i.v. pentobarbital sodium administration (200 mg/kg BW). Euthanasia was performed in accordance with the ethical standards of the institutional Animal Care and Use Committee and were approved by the committee. Isolation of adipocytes from the stromal-vascular (SV) cells was performed according to Rodbell [15] and modified by Etherton et al. [16]. Three isolations were performed from 3 different crossbred female pigs (Yorkshire × Landrace × Poland China). Adipocyte and SV cell fractions were then extracted for total RNA.

Pigs younger than 21 d of age could not be used for this experiment as the adipocyte isolation procedure depends upon separation of adipocytes from SV cells by flotation. Adipocytes from younger pigs are too small and therefore insufficiently buoyant to adequately separate a sufficient number from the SV cell populations. The net result of these procedures was adipocyte fractions and SV cell fractions for each of three 21 d old pigs.

### Isolated preadipocyte/Stromal-vascular cell culture

Isolated cell cultures were used for the analysis of ORM1 regulation of cytokine mRNA abundace. Dorsal, subcutaneous adipose tissue was obtained from between the second and fourth thoracic vertebrae of three- to seven d old male, crossbred pigs (Yorkshire × Landrace × Poland China) following i.v. pentobarbital sodium administration (200 mg/kg BW). Euthanasia was performed in accordance with the ethical standards of the institutional Animal Care and Use Committee and were approved by the committee. Primary cultures containing pig preadipocytes and SV cells were prepared by conventional methods previously published [17]. Cells were seeded on six well tissue culture plates at a density of 1.0 × 10^4^ cells/cm^2^ in DMEM/F12 containing 10 % FBS (vol/vol), 10,000 U/L penicillin sodium, 100 mg streptomycin sulfate/L, 250 μg amphotericin B (plating medium). Cells were kept at 37 °C in a humidified, 5 % CO_2_ atmosphere.

Cells were maintained in plating medium until confluency (d 5 to 6 of culture), with medium changed every 2 d. At confluency, cultures were induced to differentiate and accumulate lipid using DMEM/F12 medium containing 2.5 % pig serum (Sigma, St. Louis, MO), 10 nmol/L insulin and 100 ng hydrocortisone/mL. After 7 days of lipid filling, these cell cultures containing differentiated adipocytes and SV cells were washed free of this medium, incubated overnight in DMEM/F12 containing 1 % pig serum and then used for the experiment. The next morning, this medium was washed from the cultures with serum free DMEM/F12 and replaced with treatment medium for 24 h. Treatment media were comprised of serum-free DMEM/F12 with 0.5 % dialyzed BSA, 10,000 U/L penicillin sodium, 100 mg streptomycin sulfate/L, 250 μg amphotericin B/L, 10 nmol/L insulin and supplemented with 0, 100, 1,000, or 5,000 ng porcine ORM1/mL medium. The concentrations of ORM1 used in this experiment was based upon identifying the minimal effective concentration that affects metabolism as the extreme cost of the protein prohibits the use of physiological concentrations while maintaining species specificity (homology among species is less than 70 %). Secondly, these low concentrations were used to minimize non-specific binding by ORM1 [18–20].

Porcine ORM1 derived from normal pig serum (α1-acid glycoprotein; Life Diagnostics, Inc, West Chester PA) was prepared in phosphate buffered saline, pH 7.4 at 1 mg/mL. Individual ORM1 aliquots were thawed for each day of use and diluted in incubation medium to the appropriate concentration. The experiment was repeated five times, with cell cultures derived from five separate pigs, using duplicate tissue culture wells/treatment for each pig.

Following 24 h of incubation, treatment medium was collected from wells and frozen at −80° for later analysis of TNFα and IL6 protein content. Wells were washed with Hank’s balanced salt solution, blotted and then cells were scraped and transferred in 1.0 mL Qiasol extraction solution (Qiagen, Valencia, CA) to a microfuge tube for RNA extraction.

### Absolute quantitative PCR (qPCR) for ORM1 in subcutaneous adipose tissue and liver of preweaning pigs

Absolute quantitative PCR was used to determine the number of ORM1 mRNA molecules present in subcutaneous adipose tissue and liver per nanogram of total RNA and thus permit comparisons between the two tissues. The PCR-derived ORM1 cDNA was T/A cloned into the pGEM-T Easy plasmid (Promega, Madison, WI) that was subsequently transformed into JM109 competent cells. The ORM1/plasmid DNA was purified with the Qiaprep® Spin Miniprep kit (Qiagen, Valencia, CA) according to the manufacturer’s instructions to establish a DNA stock for the standard curve of each respective gene. The plasmid DNA was linearized with Nde I or Not I restriction enzymes (ThermoFisher Scientific, Waltham, MA) based on insert orientation. Linearized plasmid was transcribed in vitro using MEGAscript kit (Life Technologies, Grand Island, NY) utilizing T7 or Sp6 RNA polymerases for Nde I and Not I linearized plasmids, respectively. Synthesized RNA was purified and concentration was evaluated on a NanoDrop 2000 Spectrophotometer (ThermoFisher Scientific, Waltham, MA). A copy number of specific RNA is sequence and length dependent and was calculated accordingly to the equation described previously [21]. A dilution series of copy number 10^11^ to 10^2^ molecules per microliter was performed in the presence of yeast tRNA at 66 μg/ml. The dilution series for the standard curve was reverse-transcribed in parallel with one microgram of total sample RNA using Superscript (Life Technologies, Carlsbad, CA) as previously described [22]. For qPCR, 7.5 ng RNA equivalents of cDNA were combined with ORM1 specific-primers (100 picomoles) in a 25 μl reaction volume and IQ™ SYBR® Green Supermix (BioRad, Hercules, CA). Amplification was performed on the CFX96 thermocycler (BioRad, Hercules, CA) with the following cycling conditions: denature; 30 s at 94 °C, annealing 30 s at 60 °C and extension; 60 s at 72 °C. The synthesis of a single PCR product was confirmed by melt curve and agarose gel analyses. ORM1 mRNA abundance in subcutaneous adipose and liver from five pigs at each of three ages (d1, d7 or d21) were quantified by this procedure. Data were expressed as molecules/ng total RNA.

### Real-time PCR analysis of mRNA abundance in isolated cell fractions and cell cultures

Total RNA was isolated using Qiagen RNeasy spin columns according to the manufacturer’s protocol (Qiagen). Integrity of RNA was assessed via agarose gel electrophoresis and RNA concentration was determined spectrophotometrically using A260 measurements.

All primer sets were designed to span an intron as previously described and utilized for real-time PCR (Table 1), [13, 17, 23]. The specific genes of interest were selected based upon their reported roles in the inflammatory response within adipose tissue [12]. Cyclophilin was used as the reference gene [24].

**Table 1:** Primer sequences for PCR of porcine adipokines

Gene	Primer	Primer Sequence	Amplicon Size, bp	Amplification Efficiency, %	GenBank Accession No.
Tumor Necrosis Factor α	Sense	5'-CCCCTCTGAAAAAGACACCA-3'	180	106	NM214022
	Antisense	5'-TCGAAGTGCAGTAGGCAGAA-3'			
Interleukin 6	Sense	5'-ATGGCAGAAAAAGACGGATG-3'	215	98	NM214399
	Antisense	5'-GTGGTGGCTTTGTCTGGATT-3'			
MCP (*CCL2*)	Sense	5'-TCTCCAGTCACCTGCTGCTA-3'	210	95	NM214214
	Antisense	5'-AGGCTTCGGAGTTTGGTTTT-3'			
MIF	Sense	5'-CGATGTTCGTGGTAAACACC-3'	235	101	DQ989235
	Antisense	5'-AGCAGCTTGCTGTAGGAACG-3'			
Adiponectin	Sense	5'-TACCCCAGGCCGTGATGGCA-3'	213	92	AY135647
	Antisense	5'-ACCCGAGTCTCCAGGCCCAC-3'			
Cyclophilin	Sense	5'-ATGGTAACCCCACCGTCTTC-3'	376	99	AY008846
	Antisense	5'-GTTTGCCATCCAACCACTCAG-3'			

*MCP* monocyte chemotactic protein, *MIF* macrophage migration inhibitory factor

Thermal cycling and data acquisition were performed with a Bio-Rad iCycler iQ system (Bio-Rad Laboratories Inc., Hercules, CA). Reverse transcription (RT) and real time PCR analysis were performed in a two tube assay as previously described [13, 17]. Reverse transcription was done using a SuperScript First-Strand Synthesis System for RT-PCR (Invitrogen, Carlsbad, CA) according to the manufacturer’s directions.

Real time PCR was done using the IQ Sybr Green Supermix kit (Bio-Rad Laboratories Inc.) according to the manufacturer’s directions. Parameters for all reactions were as follows: 1 cycle at 95 °C for 15 min, followed by 30 cycles, 94 °C for 15 s, 58 °C for 30 s, 72 °C for 30s, with a final extension at 72 °C for 8 min. Melting curve analysis was performed on all real time PCR reactions to confirm specificity of the real time PCR products. Specificity of real time PCR products was further confirmed by agarose gel electrophoresis.

### Quantification of mRNA abundance

At the end of the PCR, baseline and threshold crossing values (Cq) for all analyzed genes were calculated using the BioRad software and the Cq values were exported to Microsoft Excel for analysis. The relative expression of the genes of interest, standardized against the amount of cyclophilin mRNA, was calculated using the ∆∆CT method [25, 26]. Values were calculated as the mean ± SEM of duplicate determinations from duplicate tissue culture wells derived from each of five individual animals. Data are expressed relative to values determined for incubations without ORM1, whose value was arbitrarily set at 1.0.

### ELISA analysis of porcine TNFα and IL6

Enzyme-linked immunosorbent assay (ELISA) kits specific for pig TNFα and IL6 were used (R&D Systems, Minneapolis, MN). Frozen medium was thawed, centrifuged for 2 min at 21,000 × g in a microfuge and then a 50 μL aliquot was used for the ELISA according to the manufacturer’s protocol. Intra-assay CV was less than 6.2 % for TNFα and less than 5.1 % for IL6 according to the manufacturer’s instructions. Inter-assay CV was less than 10.0 % for TNFα and less than 7.4 % for IL6 according to the manufacturer’s instructions. Values were calculated as the mean ± SEM of duplicate determinations from duplicate tissue culture wells derived from each of four individual animals. Data are expressed as pg/mL/24 h.

### Statistical analysis

Data were analyzed by analysis of variance using SigmaPlot 12 software (SPSS Science, Chicago, IL) to test for the effects of treatment with ORM1. Mean separation was analyzed using Student-Newman-Keuls test. If Shapiro- Wilk normality test determined the data were not normally distributed, then Kruskal-Wallis one way ANOVA on ranks was performed and all pairwise comparisons were evaluated with Tukey’s test. Means were defined as different at *P* < 0.05.

## Results

**Fig. 1:**
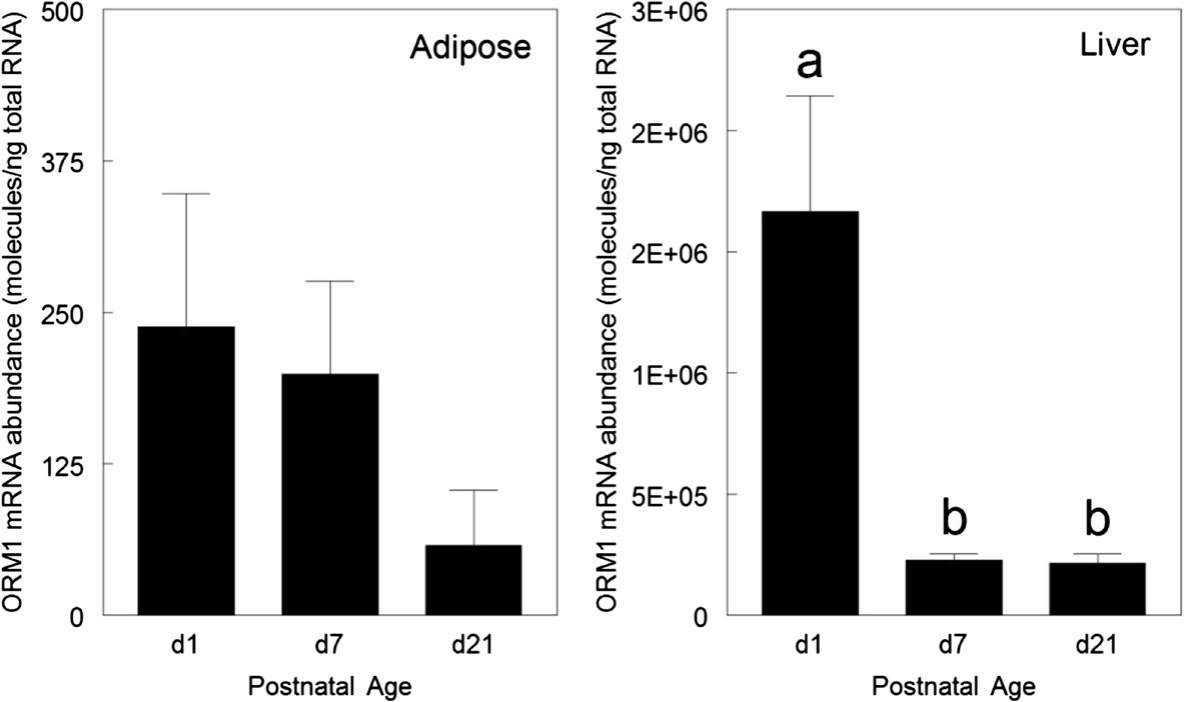
Ontogeny of ORM1 expression in the liver and subcutaneous adipose tissue of the preweaning pig. Dorsal subcutaneous adipose tissue and liver were collected at d 1, 7 or 21 of age. Different pigs from different litters were used at all time points. Tissue samples were frozen in liquid nitrogen for later analysis of ORM1 by absolute quantitative PCR. The Y axis represents number of copies of ORM1 mRNA per nanogram of total RNA as described in the methodology. The standard curve for liver samples had a slope of −3.307 and y intercept of 39.674 with an *R* ^2^ = 0.984. The standard curve for adipose tissue samples had a slope-3.363 and y intercept of 39.856 with an *R* ^2^ = 0.996. ^ab^ Means not sharing a common superscript letter are different (*n* = 5; *P* < 0.05)

The number of molecules of ORM1 mRNA/ng total RNA in liver was many thousand-fold higher than in the subcutaneous adipose tissue of the preweaning piglet at any of the three ages examined (Fig. 1). Adipose tissue ORM1 mRNA abundance did not change from d 1 to 21 of age (*P* > 0.05); although the level was extremely small. However, ORM1 mRNA abundance in liver decreased 86 % between d 1 and 7 of age (*P* < 0.001), but did not change significantly between d 7 and 21 (*P* > 0.05).

**Fig. 2:**
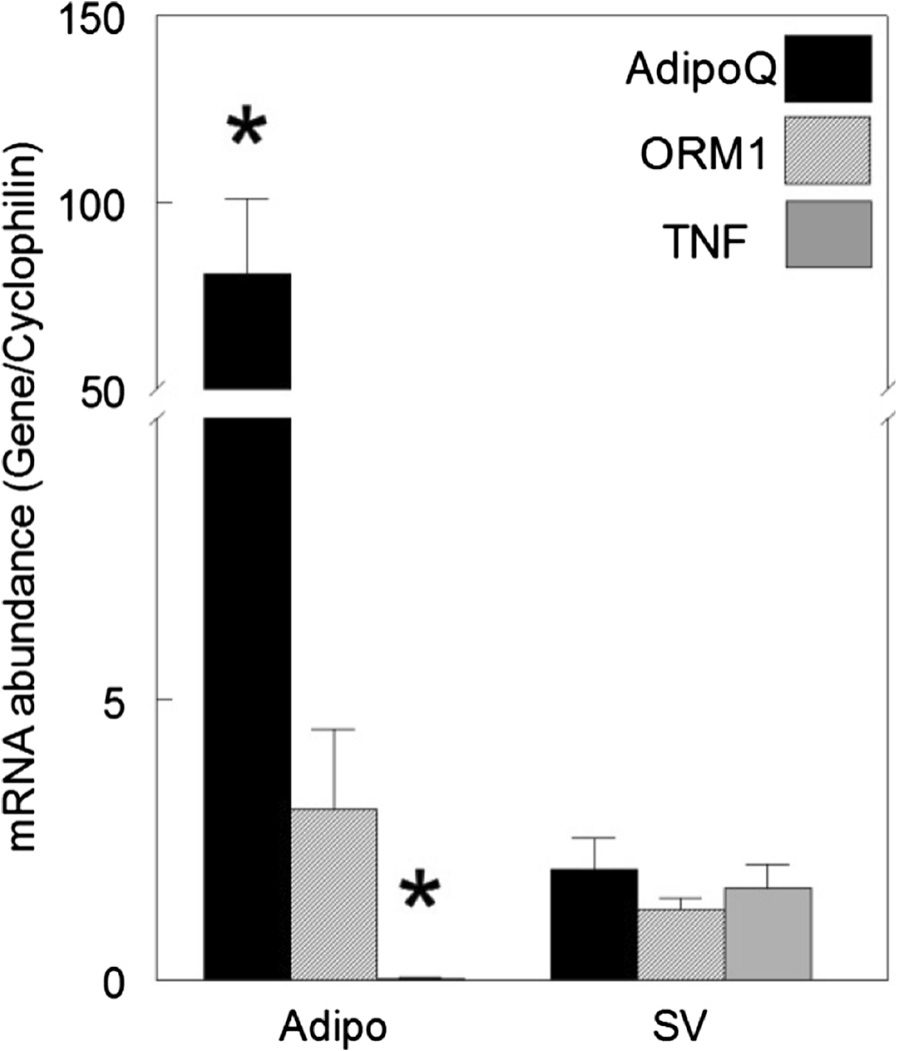
The mRNA abundance of ORM1 relative to cytokines in isolated adipocytes (Adipo) and stromal-vascular cells (SV). Cells were isolated from 21 d old pigs (*n* = 3 pigs) by collagenase digestion, filtration and flotation as described in the methodology, prior to RNA extraction and RT-PCR for relative quantification of adiponectin (adipoQ), ORM1 and tumor necrosis factor α (TNF). The mRNA abundance data are expressed relative to cyclophilin using the ∆∆CT method as described in the methodology. ^*^ Different from mRNA abundance in SV cells (*n* = 3; *P* < 0.05)

The mRNA abundance of ORM1 in the adipocyte fraction of cells derived from subcutaneous adipose tissue of 21 d pigs was similar to the abundance in the SV cells (Fig. 2, *P* > 0.05). As expected, adiponectin (AdipoQ) was expressed primarily by the adipocyte fraction, with SV cells expressing only approximately 2.4 % of the level found in adipocytes (*P* < 0.001) thus confirming cell separation and identity. In contrast, the mRNA abundance for TNFα was 56-fold higher in the SV cells than in the adipocytes isolated from the same tissue (*P* < 0.001).

**Fig. 3:**
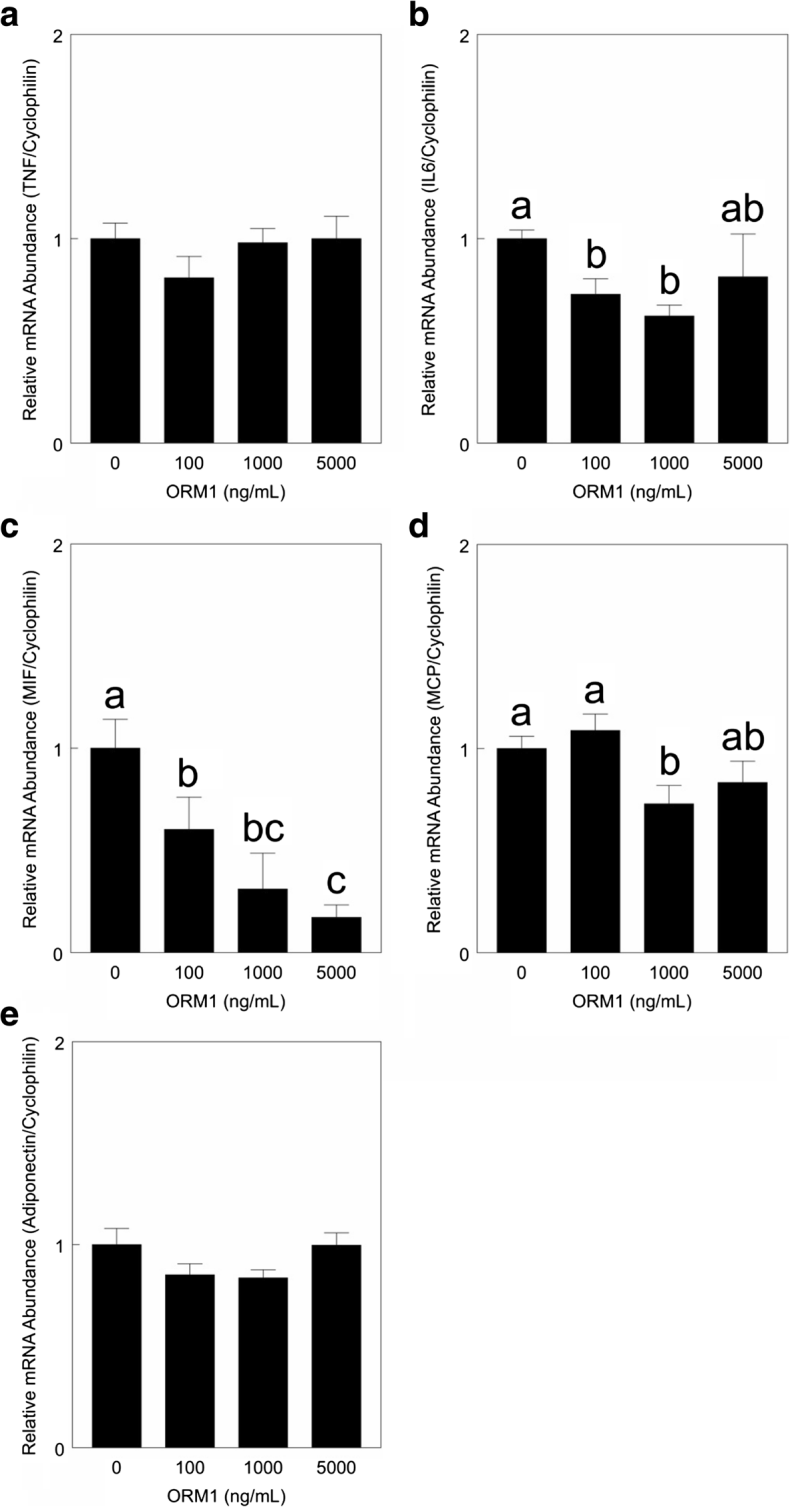
Relative mRNA abundance for cytokines in primary adipose cultures in response to ORM1 treatment. Primary cultures were derived from neonatal porcine subcutaneous adipose tissue according to procedures described in the Methods section. Cultures containing differentiated adipocytes and stromal-vascular cells were incubated with 0, 100, 1000 or 5000 ng porcine ORM1/mL medium for 24 h. Relative tumor necrosis factor α (TNF, **a**), interleukin 6 (IL6, **b**), macrophage migration inhibitor factor (MIF, **c**), monocyte chemotactic protein (**d**) and adiponectin (**e**) mRNA abundance were measured by real time PCR as described in the methodology. The relative expression of the genes of interest, standardized against the amount of cyclophilin mRNA in each cell culture was calculated using the ∆∆CT method. Data are expressed relative to values determined for incubations without ORM1, setting these values to 1. ^abc^ Means not sharing a common superscript letter are different (*n* = 5; *P* < 0.05)

Incubation of primary cultures containing differentiated adipocytes and SV cells derived from neonatal adipose tissue with increasing concentrations of porcine ORM1 for 24 h had no effect on TNFα mRNA abundance (*P* > 0.05, Fig. 3a). In contrast, treatment with ORM1 reduced IL6 mRNA abundance at 100 and 1,000 ng ORM1/mL medium by approximately 32 % (*P* < 0.01, Fig. 3b). The MIF mRNA abundance in these primary cultures was reduced in a dose responsive manner, with a maximal reduction of 83 % at 5000 ng ORM1/mL medium (*P* < 0.01; Fig. 3c). An effect of ORM1 treatment on MCP mRNA level was only detectable at a concentration of 1000 ng ORM1 /mL medium (*P* < 0.05, Fig. 3d). Adiponectin mRNA abundance was not affected by incubation with ORM1 in primary cultures derived from neonatal adipose tissue (*P* > 0.05; Fig. 3e).

**Fig. 4:**
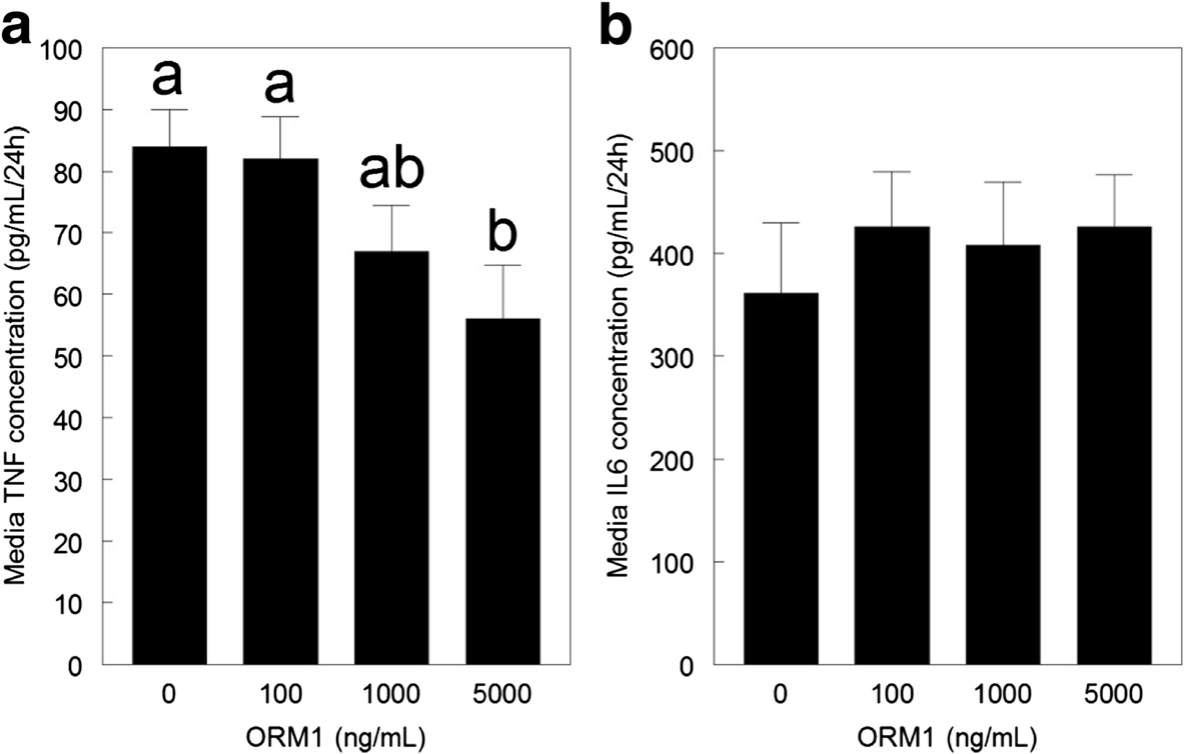
Cytokine content in medium from primary adipose tissue cultures in response to ORM1 treatment. Primary cultures were derived from neonatal porcine subcutaneous adipose tissue according to procedures described in the Methods section. Cultures containing differentiated adipocytes and stromal-vascular cells were incubated with 0, 100, 1000 or 5000 ng porcine ORM1/mL medium for 24 h. The TNF (**a**) and IL6 (**b**) protein concentrations in the media were measured by ELISA as described in the methods. Data are expressed as pg/ml medium/24 h. ^ab^ Means not sharing a common superscript letter are different (*n* = 4; *P* < 0.05)

The medium concentration of TNFα was reduced by incubation of primary cultures of differentiated adipocytes and SV cells with ORM1. This decrease was approximately 33 % at 5000 ng/mL medium (*P* < 0.05; Fig. 4a). Medium IL6 concentration was not affected by treatment of cell cultures with ORM1 (*P* > 0.05, Fig. 4b).

## Discussion

Orosomucoid represents the major circulating protein in the serum of newborn pigs [5, 6]. Serum concentrations of ORM1 in the newborn pig vary from 8,000 to 12,000 μg/mL and decline rapidly after birth [6, 27], which parallels the observed changes in mRNA abundance in the liver in the present study. However, adipose tissue ORM1 mRNA abundance was extremely low relative to the liver and at the absolute lowest limits of detection. These data suggest that adipose tissue makes minimal contribution to the high ORM1 concentrations in the serum of the preweaning piglet. This does not exclude the adipose derived ORM1 from functioning in the paracrine environment of the adipose tissue in vivo.

The cell source for any paracrine ORM1 of adipose origin appears to include both adipocytes and the SV cells based upon the similar ORM1 mRNA abundance between the two cell populations isolated from porcine adipose tissue. The quality of the separation of the two cell populations was confirmed by the analysis of the expression of adiponectin and TNFα in the same isolated cell populations. Fain et al. [28] reported that adiponectin is expressed primarily by the adipocyte fraction from human adipose tissue, while TNFα is expressed almost exclusively by the SV cells [29]. Similar results were found in the present study for the distribution of adiponectin and TNFα mRNAs between pig adipocytes and SV cells.

Addition of porcine ORM1 to primary cultures containing adipocytes and SV cells derived from neonatal pig adipose tissue had no effect on TNFα mRNA abundance. This is in disagreement with Lee et al. [30] who reported that human ORM1 induced a two-fold reduction in TNFα mRNA abundance in murine 3 T3-L1 adipocytes. This may be due to a species difference or an effect of differences in cell culture methodology. Lee et al. [30] used human ORM1 at a concentration of 250 μg/mL medium with the murine 3 T3-L1 murine cell line versus the much lower concentrations of porcine ORM1 used in the present study (100–5,000 ng/mL) while maintaining species specificity with the porcine adipocytes and SV cells. This suggests a much higher concentration of ORM1 may be necessary to elicit a change in TNFα mRNA abundance than used in the present study.

The analysis of the media content of TNFα following 24 h of incubation with ORM1 suggests that ORM1 may regulate TNFα post-transcription. The reduction in TNFα accumulation in the medium occurred while TNFα mRNA abundance was not affected by ORM1. Since the observed reduction was relatively small, some caution must be used in the interpretation. Previous research has demonstrated that TNFα can interfere with insulin mediated glucose metabolism in neonatal porcine adipose tissue [17], thereby creating an insulin resistant state that is characteristic of adipose tissue inflammation in obesity, diabetes and metabolic syndrome [31]. The present data suggest that ORM1 does not contribute to adipose tissue inflammation in the pig at the low concentrations tested by stimulating TNFα expression or secretion, despite these same ORM1 concentrations (1 and 10 μg/mL medium) reducing glucose utilization by the tissue in vitro [14].

TNFα can regulate the expression of a number of cytokines within neonatal porcine adipose tissue, including stimulating the expression of the pro-inflammatory cytokines IL6, MIF and MCP [17]. A reduction in IL6 mRNA level by ORM1 has been previously reported for murine 3T3-L1 adipocytes [30]. The change in IL6 mRNA abundance was relatively small in the present study, so it is not surprising that no effect of ORM1 treatment was detected on the secretion of IL6 into the medium of cultures derived from neonatal pig adipose tissue. This suggests that IL6 does not contribute to the effect of ORM1 to reduce insulin stimulated glucose utilization in neonatal porcine adipose tissue, despite using the same low concentrations of ORM1 (14). This does not exclude the possibility that higher and physiological concentrations could elicit an IL6 response similar to observed in murine 3 T3-L1 adipocytes treated with human ORM1 at 250 μg/mL [30].

Atsumi et al. [32] demonstrated that MIF reduces glucose uptake and attenuates insulin sensitivity in mouse adipocytes; thereby indicating MIF has a role in the adipose tissue inflammation reported for metabolic syndrome and obesity [31]. The dose responsive reduction in the MIF mRNA level to ORM1 treatment occurred at ORM1 concentrations beginning at 100 ng/mL medium; whereas a small decrease in TNFα release was detected at the 5,000 ng ORM1/mL medium. Thus, ORM1 may have a direct effect on MIF gene expression or may be producing this reduction in MIF through another intermediary other than TNFα, suggesting that ORM1 may have some specific anti-inflammatory effects within adipose tissue. The very high serum concentrations of ORM1 in the neonatal pig (8–10 mg/mL in the newborn [27]) suggest that ORM1 may have a significant role in regulating MIF expression within the neonatal adipose tissue in vivo, but this requires further investigation.

Adiponectin is considered an anti-inflammatory cytokine due to its numerous functions to reduce the inflammatory response, including reducing the expression of the pro-inflammatory cytokines TNFα, IL6, MCP and IL1β as well as vascular adhesion molecules and scavenger receptors in various tissues [33, 34]. The present data suggest that adiponectin may not have a role in countering ORM1 induced insulin resistance in neonatal pig adipose tissue in vitro since ORM1 did not alter adiponectin mRNA abundance.

One cannot exclude the possibility of the culture methodology itself affecting cell development, which could limit the responsiveness of the cultured cells to ORM1 and thus produce the small changes observed in mRNA abundance for IL6, MIF and MCP or the lack of changes in TNFα and adiponectin gene expression. Alternatively, the very low concentrations of ORM1 tested may be insufficient to elicit a larger, physiological response by these genes. However these same culture conditions and ORM1 concentrations have previously been used to show that ORM1 can reduce the mRNA abundance for insulin signaling proteins and lipogenic enzymes in neonatal pig adipose tissue [14]. Insulin sensitivity of adipose tissue is reduced with elevated concentrations of various inflammatory cytokines in vitro [35], but the present study suggests that the previously reported reduction in insulin stimulated glucose utilization by low concentrations of ORM1 does not involve increases in the expression of TNFα, IL6, MCP or MIF. Therefore, the mechanism of action for low concentrations of ORM1 to reduce insulin response does not appear to be through these inflammatory cytokines; but whether the mechanism is by interaction with insulin binding or glucose transport requires further investigation.

## Conclusions

Adipocytes and stromal-vascular cells from preweaning pigs contain ORM1 mRNA in similar quantities, implying both cell populations contribute to the overall pool of ORM1 produced within adipose tissue. However, ORM1 mRNA abundance in adipose tissue is extremely small compared to liver. Therefore, adipose tissue probably makes minimal contribution to circulatory ORM1 and the effects of adipose derived ORM1 in peripheral tissues are probably very limited. Addition of porcine ORM1 to primary cultures derived from adipose tissue produced limited responses by inflammatory cytokine genes and none of the genes responded with an increase in expression. These data suggest that ORM1 does not reduce insulin stimulated glucose metabolism within adipose tissue in vitro as the result of ORM1 induced paracrine expression of TNFα and related inflammatory cytokines.
